# Characterization of medical device constituents and development of duration-based non-cancer threshold of toxicological concern values

**DOI:** 10.3389/ftox.2025.1600127

**Published:** 2025-06-04

**Authors:** Taylor Builee, Todd A. Kennedy, Valériane Levelut, Megan A. Hahn, Stephen Bond, Michael K. Peterson, Frances K. Hsia, Alessia Stornetta, Kristin J. Erickson, Kimberly D. Ehman, Bindu Prabhakar, Bradford D. Bagley, Sherry P. Parker

**Affiliations:** ^1^ Toxicology Consultant, Princeton, NJ, United States; ^2^ W.L. Gore and Associates, Inc., Flagstaff, AZ, United States; ^3^ NAMSA, Biological Safety and Consulting, Chasse-sur-Rhône, France; ^4^ NAMSA, Biological Safety and Consulting, Northwood, OH, United States; ^5^ RMQ+, Jordi Labs, Mansfield, MA, United States; ^6^ Gradient, Seattle, WA, United States; ^7^ Boston Scientific Corporation, Preclinical Sciences, Arden Hills, MN, United States; ^8^ WuXi AppTec, St. Paul, MN, United States; ^9^ Solventum, Product Stewardship, St. Paul, MN, United States; ^10^ SParker Toxicology Consulting LLC, Davie, FL, United States

**Keywords:** threshold of toxicological concern, medical device, less than lifetime, fit for purpose, non-cancer

## Abstract

**Introduction:**

In the absence of sufficient constituent-specific dose-response toxicity data, threshold of toxicological concern (TTC) values are commonly used in toxicological risk assessment of medical device (MD) constituents. When experimental data or predictions suggest that a constituent is not likely to have genotoxic effects, categorizing the constituent into its appropriate Cramer Class and application of the corresponding TTC value is recommended. This paper presents the uniqueness of the MD chemical space when compared to the historical Munro TTC dataset via structure-based chemical taxonomy, ToxPrint chemotypes, physicochemical properties and molecular descriptors, and proposes duration-based MD non-cancer TTC values.

**Methods:**

More than 15,000 MD constituents were identified and screened, and 790 constituents met the established criteria for inclusion. Constituents with chemotypes matching inorganic substances, metals, pharmacologically active, nitroso-like, aflatoxin-like, azoxy, benzidine, polyhalogenated dibenzodioxins, dibenzofurans, biphenyls, high molecular weight polymers, nanomaterials, proteins, and radioactive substances were excluded from the evaluation. Constituent-specific toxicity data were obtained from the data-rich and open-access, European Chemicals Agency Registration, Evaluation, Authorisation and Restriction of Chemicals (ECHA REACH) database. Considered protective for systemic, developmental, and reproductive toxicity, constituent-specific oral no-observed-adverse-effect-level (NOAEL) values from repeated dose studies with a reliability (Klimisch) score of 1 or 2 were selected as the point of departure (POD) for each duration (subacute/subchronic/chronic/lifetime). The NOAEL values selected as PODs for each constituent in each duration category were plotted using log-normally fitted cumulative frequency distributions, and an uncertainty factor of 100 (10 each for inter and intraspecies differences) was applied to the lowest fifth percentile NOAEL value extrapolated from each curve.

**Results:**

The resulting non-cancer TTC values for various exposure duration categories were 112 μg/kg/day for ≤ 1 day to 30 days, 111 μg/kg/day for 31 to 365 days and 41 μg/kg/day for ≥ 366 days.

**Discussion:**

The proposed MD non-cancer TTC values followed the same approach as derivation of the Munro TTC values; however, they are derived exclusively from MD constituents with chemical-specific data for the appropriate period of assumed exposure to the constituent.

## Introduction

The Threshold of Toxicological Concern (TTC) is an exposure level of negligible toxicological risk applied in the assessment of constituents with limited toxicological data. The TTC concept originated from United States Food and Drug Administration (US FDA)’s Threshold of Regulation in 1995 (FDA, 1995) using an analysis of animal derived carcinogenicity data to address indirect food contact substances. The TTC is the *de facto* assessment tool applied to data-poor constituents in a wide range of regulatory contexts, with regulatory acceptance in food safety, cosmetics, fragrances, pharmaceutical, ecological, and medical device (MD) applications. TTC values have been derived using two main approaches: cancer potency-derived TTC values associated with a specific lifetime cancer risk level, typically one in a million risk level or 1 in 100,000, and non-cancer TTC values stratified according to the Cramer decision tree system with a safety factor of 100 applied to the cumulative fifth percentile point of departure (POD) of a large dataset. US FDA Center for Food Safety and Applied Nutrition (CFSAN) (2021) FDA (2021) and Patlewicz et al. (2022) demonstrated that Cramer TTC values require a fit-for-purpose (FFP) consideration based upon the chemical space in which the concept is applied. Other groups have developed FFP TTC values for the assessment of food-related substances (Reilly et al., 2019; Munro et al., 1996), cosmetics (Yang et al., 2017), fragrances (Patel et al., 2020), and botanical extracts (Kawamoto et al., 2019).

Considering the diversity of MDs and their applications, the authors have presumed that MD constituents (i.e., materials, additives, residues, degradation products, impurities, or contaminants) form a unique chemical space. However, there is limited knowledge on the collective universe of MD constituents reported in analytical studies used for regulatory purposes. Characterization of the MD constituent universe would help to understand the applicability of non-cancer TTC values as referenced in ISO/TS 21726:2019 (ISO/TS 21726, 2019) that are used for assessment of MD constituents. In addition, collection of toxicity data for MD constituents would allow for the derivation of MD-specific non-cancer TTC values, which are not listed in the first edition of ISO/TS 21726:2019 (ISO/TS 21726, 2019). Finally, current non-cancer TTC values referenced in ISO/TS 21726:2019 have been established for lifetime exposure and are used for assessing whether less-than-lifetime (LTL) exposure will be at a tolerable level, in the absence of available LTL non-cancer TTC values. While this approach can be seen as conservative, it can lead to equivocal toxicological risk assessments that over-estimate the patient risk and ultimately lead to a need for (unnecessary) animal testing.

To address these gaps, a collaborative effort was undertaken by a group of MD toxicologists to develop FFP non-cancer TTC values aligned with the duration-based framework of ISO 10993-17:2023 (ISO 10993-17, 2023). For this purpose, MD constituents were solicited from world-wide contract research organizations, MD manufacturers, and consultant groups to generate a constituent database (DB). The accuracy of constituent identifiers was verified with free and subscription-based chemical data repositories to prepare a set of constituents with unique Chemical Abstracts Service Registry Number (CAS No.) and Simplified Molecular-Input Line-Entry System (SMILES) code. Then, chemical-specific oral no-observed-adverse-effect-levels (NOAELs) from a toxicology data repository were selected based on quality assessment of the testing and duration of the study period. The TTC values were derived from the fifth percentile of the cumulative distributions of these NOAELs. These duration-based non-cancer TTC values are not applicable to highly potent substances (i.e., with a tolerable intake below 0.025 μg/kg/day or 1.5 µg/day for a 60-kg person, a generic threshold known as the US FDA Threshold of Regulatory Concern) and those defined as “excluded compounds” because these substances were not included in the dataset used to derive these values.

## Materials and methods

### Curation of a dataset of non-genotoxic medical device constituents

#### Medical device constituent dataset

Over 15,000 MD constituents (CAS No. and name) were identified by toxicologists from MD manufacturers, contract research organizations, and consultant groups from France, Italy and the U.S.A. A majority of these constituents were identified from MD extractables studies conducted in compliance with ISO 10993-18 (ISO 10993-18, 2020). No attempt was made to verify that the reported constituents were identified correctly in the extractables studies, as this information was unavailable and beyond the scope of this project. The providers of constituents to the DB wish to remain anonymous due to confidentiality agreements, but their identifications and affiliations to the MD industry has been verified by the authors. An additional set of constituents obtained from literature sources consisting of dental constituents and chemicals from the Extractables and Leachables Safety Information Exchange (ELSIE) DB were merged with the larger data set (Van Landuyt et al., 2007; Van Landuyt et al., 2011; Schmalz, 2009; Masuda-Herrera et al., 2022). The reported compound identities [i.e., CAS No. and SMILES (isomeric or canonical)] were cross-referenced from CAS SciFinder (developed by Chemical Abstracts Service, a division of the American Chemical Society) (RRID:SCR_004558), US EPA CompTox Chemicals Dashboard and PubChem (RRID:SCR_004284). The chemical identification criteria for inclusion required a CAS No., SMILES, molecular weight (MW) < 1,000 g/mol, and availability of a specific compound structure in CAS SciFinder. Unknown or Variable composition, Complex reaction products or Biological materials (UVCB) do not correspond to a specific, unambiguous chemical structure and resulted in exclusion based on inability to assign a SMILES. CAS Nos. associated with mixtures of racemic isomers (e.g., R/S, cis/trans) and constituents containing counterions (e.g., sodium salts) remained in the DB. Inorganics and metal-containing constituents (e.g., elemental, ionic, or organometallic compounds) were removed during DB curation or when identified via chemotype assessment. In some cases, the constituent’s name was changed to align with the CAS SciFinder identification (see Table 1).

**TABLE 1:** Chemical and toxicological study inclusion criteria for the TTC medical device database.

Parameter	Criteria
Chem ID	CAS No. (including mixtures of racemic isomers and compounds with counterions permissible) well defined and unique, non UVCB CAS No., SMILES (canonical and isomeric)
Chem properties	Molecular weight < 1,000 g/mol
Chem structure	Distinguished by CAS SciFinder
Reference	CAS SciFinder software
Chem classification	Absence of ToxPrint chemotypes associated with excluded chemical groups
Genotoxicity	Non-genotoxic experimental data or negative QSAR genotoxic prediction via ToxTree Toxtree Benigni/Bossa: No VEGA QSAR: Non-mutagenic and Applicability Domain Index (ADI) > 0.75
Study Type	Repeat dose: short term, subacute, subchronic, chronic, chronic/carcinogenicity, systemic/reproductive, reproductive/developmental, developmental, 1-Gen, 2-Gen
Reliability	Klimisch score ≤ 2
Species	Rodents (rat, mouse hamster), rabbits, dogs, non-human primates, other non-human species
Treatment duration	subacute (14–28 days), subchronic (∼60–90 days), chronic (>90–180 days), and lifetime (>1.5–2 years)
Route of exposure	Oral administration only: gavage, diet, drinking water
Reference	ECHA REACH database study summary

Genotoxicity and quantitative structure–activity relationship (QSAR) Assessment Determination of DNA reactivity was supported by application of QSAR models and experimental data sourced from the European Chemicals Agency Registration, Evaluation, Authorisation and Restriction of Chemicals (ECHA REACH) DB. Data obtained from testing conducted according to or similar to the Organisation for Economic Cooperation and Development (OECD) Guidelines for the Testing of Chemicals, Section 4 (Health Effects), such as the bacterial reverse mutation (mutagenicity) assay (471), the mouse lymphoma assay (490) or the *in vivo* micronucleus test (474) were reviewed to determine if there were a genotoxic mode of action. Predictive genotoxicity assessment was performed by expert rule-base and statistical-based QSAR models using ToxTree (v. 3.1.0) (RRID:SCR_012086), Benigni/Bossa model for mutagenicity and VEGA (v. 1.2.3) CAESAR mutagenicity model (v. 1.0.3), respectively. Consideration was given to the Benigni/Bossa output, as well as the CAESAR prediction when the Applicability Domain Index (ADI) was greater than 0.75. The ADI range of 1 to 0.85 and between 0.85 and 0.75 provided a high to moderate (respectively) prediction classification of probable reliability (Danieli et al., 2023). High priority was given to experimental genotoxicity mutagenicity and/or clastogenicity data obtained from the ECHA REACH DB and superseded QSAR predictions. ECHA REACH DB conclusions noted as positive for genotoxicity (results for the CAS No., from read-across to related compound(s), or from a weight of evidence approach); equivocal for genotoxicity; or QSAR results indicating possible genotoxicity resulted in the constituent being removed from the DB and not analyzed further (see Table 1).

The ToxTree Revised Cramer module was used to generate the Cramer Class (I, II, III) prediction. The ToxTree Revised Cramer module represents a combination of the original Cramer scheme and industry provided data on metabolism, toxicity, and biochemistry of compounds in the dataset. Additionally, Cramer decision tree scheme Q1 and Q22, which require lists of common body constituents and common components in food, respectively, are prepopulated in the software to aid in reducing variability of scoring (Cramer et al., 1978; Patlewicz et al., 2008).

#### Compound exclusion classification

A list of chemical categories was used to exclude certain constituents from the calculation of TTC values. Such lists are commonly referred to as the cohort of concern or exclusion lists. These lists describe structure-based categories that contain a significant proportion of highly potent toxicants (e.g., aflatoxin-like) or are poorly represented in the toxicity database used to derive historical TTC values (e.g., proteins) (ISO/TS 21726, 2019; More et al., 2019). Here, the list is referred to as the exclusion list. The exclusion list closely follows that from the most recent European Food Safety Authority (EFSA) TTC guidance (More et al., 2019). The chemical categories are aflatoxin-like; azoxy; N-nitroso; polyhalogenated–dibenzodioxins, -dibenzofurans, and -biphenyls; steroids; benzidines; metals in elemental, ionic, or organic form (except salts where counter ion is essential, such as sodium); high molecular weight polymers (HMWP) (>1,000 g/mol); nanomaterials; inorganic substances; proteins; radioactive constituents; and substances with known or suspected pharmacological activity. Boron-containing substances were considered inorganic and were therefore excluded. Organosilicon substances were not excluded. Other silicon-containing substances were considered inorganic and were excluded.

The dataset was analyzed using ChemoTyper software (version 1.0, rev. 12976) (Yang et al., 2015), which identifies chemical structural fragments based on SMILES. ToxPrint chemotypes were selected by generating SMILES for several chemicals listed in Appendix 1 of Cheeseman et al. (1999), that correspond to the above-mentioned categories and running those against the ToxPrint_v2.0_r212.xml file. The chemicals listed in Appendix 1 were selected manually based on their structures and how well they represented the exclusion categories described above. Those chemotypes were used to create a separate xml file. (See Supplementary File 1 for a list of chemotypes that were used.) To verify that the xml file operated as intended, the chemicals listed in Appendix 1 of Cheeseman et al. were processed with Chemotyper using the xml file and were classified correctly. The SMILES for certain organic metallics were not able to be processed by ChemoTyper. These structures were identified to be excluded readily by the presence of a metal atom. Identification of excluded substances was also verified manually. Pharmacologically active substances were identified *ad hoc* based on name and information found in publicly available databases such as PubChem (accessed between 2023-2024).

### Selection of the points of departure

#### Selection and use of a toxicological database for data collection

Constituent-specific toxicity data were obtained from the ECHA REACH database, available at: https://echa.europa.eu/fr/information-on-chemicals (ECHA, 2024a). ECHA is an Agency governed by European Union (EU) public law, which works together with the European Commission, the European Parliament, the Council of the European Union and other European Union (EU) agencies (ECHA, 2024b). The ECHA chemicals DB was selected for data collection because it is data-rich and contains open-access information from all REACH registrations received by the Agency. The ECHA REACH database is commonly used to obtain information for the evaluation of MD extractables, as toxicological information for these constituents is often lacking in other publicly available databases.

REACH is an EU regulation that intends to protect human health and the environment from the risks that can be posed by chemicals in products marketed within the European Union. REACH establishes procedures for collecting and assessing information on the properties and hazards of substances. To comply with the regulation, companies must identify and manage the risks linked to the substances they manufacture and market in the EU. Article 10 of REACH outlines the minimum information that must be submitted as part of a registration, with requirements generally increasing with increasing tonnage manufactured or imported. For example, 28-day repeated dose studies and reproductive/developmental toxicity screening tests are required at the lower end, whereas 90-day repeated dose toxicity studies and pre-natal developmental toxicity studies are required as tonnage increases. Testing is not required for carcinogens and germ cell mutagens for which risks are controlled. Factors that can influence the testing requirements are QSARs, mutagenic and carcinogenic properties, available data from humans exposed to the substance and concerns for endocrine disruption. According to Article 13(3) of REACH, tests required for generating information on substances shall be conducted in accordance with the test methods included in a Commission Regulation or in accordance with other international test methods recognised by the Commission or the Agency as being appropriate. Toxicological and ecotoxicological tests and analyses shall be carried out in compliance with the principles of Good Laboratory Practice (GLP). ECHA receives and evaluates individual registrations for their compliance, and the EU Member States evaluate selected substances to clarify initial concerns for human health or for the environment (ECHA, 2024c; ECHA, 2011).

#### Review of toxicological data

For each constituent of the DB, available toxicity data from its REACH registration dossier were searched from the ECHA website between July 2023 and July 2024, using the “Search for chemicals” function and entering the CAS No. (*N.B*.: at the time of the data collection, the new ECHA CHEM database was not yet released). Information contained in the Registered Substances Factsheets (under “Toxicological information”) was screened for genetic toxicity (*in vitro* and *in vivo*), repeated dose toxicity, carcinogenicity and toxicity to reproduction (including developmental toxicity/teratogenicity) data. The genotoxicity data were reviewed to confirm that each constituent was negative for this endpoint.

For the repeated dose toxicity, carcinogenicity and toxicity to reproduction studies, only data from oral administration were considered, as toxicity data using this route of exposure is generally the most commonly found in REACH dossiers. Data pertaining to inhalation, dermal, intraperitoneal, and intravenous administration routes of exposure were limited and, therefore, excluded. The ECHA REACH reliability assessment of toxicity data is based on the Klimisch scoring system (Klimisch et al., 1997), which favors studies conducted per GLP and those using an accepted test guideline method. Toxicity studies were selected only if they had an assigned reliability (Klimisch) score of 1 (reliable without restrictions) or 2 (reliable with restrictions). In addition, the studies had to be CAS No.-specific (i.e., studies were conducted on the constituent itself). In addition to reviewing experimental details about the specific oral route of exposure (e.g., gavage, diet, drinking water) and noted adverse effects, information regarding study type, study duration, species, and NOAEL/lowest-observed-adverse-effect-level (LOAEL) determination were obtained from the substance’s dossier and/or the cited OECD Test Guideline that was followed. If not reported in the dossier entry, daily doses (mg/kg/day) were calculated by applying approximate conversion factors to the reported test substance concentrations from feed or drinking water (ppm), as applicable (Derelanko, 2008; EFSA, 2012). The following approximate study durations were considered: subacute (14-28-day), subchronic (90-day), chronic (180-day) and lifetime (2-year).

#### Point of departure selection

To determine MD duration-based non-cancer TTC values, a point of departure (POD) for each duration (when available) was selected for each MD constituent based on the expert judgment of experienced toxicologists and the following systematic approach, which aligns with the methodology for choosing a POD outlined in ISO 10993-17 (ISO 10993-17, 2023).

In general, the lowest NOAEL for each duration category was chosen, assuming there was at least one LOAEL reported. If there were no LOAELs reported for any study within a duration category (i.e., the reported NOAEL for each study was the highest dose tested), the highest NOAEL among the reported NOAELs was chosen as the POD for that duration, because no observed adverse effects were reported in any study.

The durations of studies for toxicity to reproduction were considered in the context of the exposure scenario in an analogous repeated dose toxicity study. When a NOAEL was established from a reproductive toxicity study, a reproductive/developmental toxicity study (e.g., standalone or combined with repeated dose systemic toxicity study) or developmental toxicity study, it was considered along with the NOAELs from the systemic toxicity studies for a particular duration. For example, if a compound had an established NOAEL from a developmental toxicity study (OECD 414, dams dosed 14 days) that was less than the NOAEL from a 28-day repeated dose systemic toxicity study (OECD 407), the lower NOAEL from the developmental toxicity study was considered for the 14-28-day duration as a conservative measure. Similarly, if an established NOAEL from an extended one-generation reproductive study (OECD 443, P0 animals dosed 10–12 weeks) was less than the NOAEL from a 90-day repeated dose systemic toxicity study (OECD 408), the lower, more conservative NOAEL was considered for the 90-day duration.

Two-year studies were either standalone chronic systemic toxicity (OECD 452), carcinogenicity (OECD 451), or combined chronic toxicity/carcinogenicity (OECD 453). Any established NOAELs based on cancer effects (i.e., cancer observed in animals at the LOAEL) were also considered, as these NOAELs were assumed to be due to non-genotoxic carcinogenicity, because genotoxic compounds were previously excluded from the analysis.

At least one other experienced MD toxicologist verified that the constituent was suitable for inclusion in the analysis (i.e., it was not genotoxic) and confirmed the POD for each duration. Using this strategy, the chosen POD (i.e., NOAEL) was protective for systemic toxicity, reproductive toxicity, developmental toxicity, and non-genotoxic carcinogenicity.

### Derivation of a non-cancer TTC value

For each duration, the selected NOAELs were plotted using log-normally fitted cumulative frequency distribution curves with the software GraphPad Prism (v.10.4.0) (RRID:SCR_002798). The lowest fifth percentile NOAEL value was interpolated from the curve and was adjusted by an uncertainty factor of 100 (10 each for inter and intra-species differences) to result in the duration specific TTC (ISO 10993-17, 2023). Upper and lower 95% confidence intervals were calculated. NOAELs from 180-day and 2-year studies were combined to achieve a larger sample size. In the combined list of 180-day and 2-year studies, for studies in dogs, only NOAELs from 2-year studies were included. In rodents, when a NOAEL was available at both durations, the lowest value was selected for the analysis.

### Chemical space analysis

The screened constituents from the MD DB were profiled using the ToxPrint chemotypes library and ChemoTyper software (v. 1.0, rev. 12976). The outcome of the substructure categorization was plotted to assess the uniqueness of the chemical space occupied by MD constituents based on analysis of frequency.

ClassyFire software (v. 1.0) was used to determine the structural classification of chemical entities. ClassyFire taxonomic reporting provided information on hierarchical chemical classification (mostly small molecules and short peptide sequences) in addition to structure-based textual description using ChemOnt, which covers 4,825 chemical classes of (in)organic compounds (Djoumbou Feunang et al., 2016). The compounds were plotted according to SuperClass levels.

The National Toxicology Program’s (NTP’s) Integrated Chemical Environment (ICE) software (v. 4.0.2) (RRID:SCR_002616) (NTP ICE, 2024), Chemical Characterization Dynamic Principal Component Analysis (PCA) was used to visualize chemical characteristics, based on a set of molecular descriptors that are mathematical representations of chemical structures (e.g., XLogP, MW, LipinskiFailures, nRotB, topoShape, nAromBond, nAcid, nHeavyAtom, nBonds, etc.). Data points that are close together on a dynamic PCA plot have similar molecular descriptors and, therefore, similar chemical structures/structural characteristics. CAS Nos. were utilized as the chemical identifiers in ICE.

An electronic record of the Munro et al. (1996) dataset with SMILES code and CAS No. (five duplicates removed) obtained from Bassan et al. (2011) was used to establish a baseline for comparison using the above methods.

## Results

### MD database metrics and analysis of the fifth percentile cumulative distribution to derive duration-based TTC values

The initial collection of more than 15,000 constituents was distilled to a subset of 1,737 chemicals with toxicological data in the ECHA REACH dossier. From this subset, another 107 constituents were removed: constituents without a structure associated to the CAS No., those that were incompletely defined, UVCBs, mixtures (except salts and racemic mixture) and polymeric substances with MW > 1,000 g/mol. This screening brought the number of constituents down to 1,630 chemicals.

In the next step, an additional 87 constituents with experimental or predictive positive or equivocal genotoxicity results were screened out. Another 58 substances were excluded because they belonged to categories in the exclusion list. The most common excluded category was inorganic substances: there were 32 such constituents. The ChemoTyper application detected five constituents comprised of a metal such as copper or chromium that were not initially excluded during DB compilation. The five metal-containing constituents were removed. There were 20 pharmacologically active substances excluded, of which seven were steroids. There was one nitroso-like substance excluded. No aflatoxin-like, azoxy, benzidine, or polyhalogenated–dibenzodioxins, –dibenzofurans, –biphenyls, HMWP, nanomaterials, proteins and radioactive substances were identified in the DB. Refer to Supplementary File 2 for the list of constituents removed from the DB due to meeting at least one exclusion criterion and Supplementary File 3 for excluded compound list..

**FIGURE 1:**
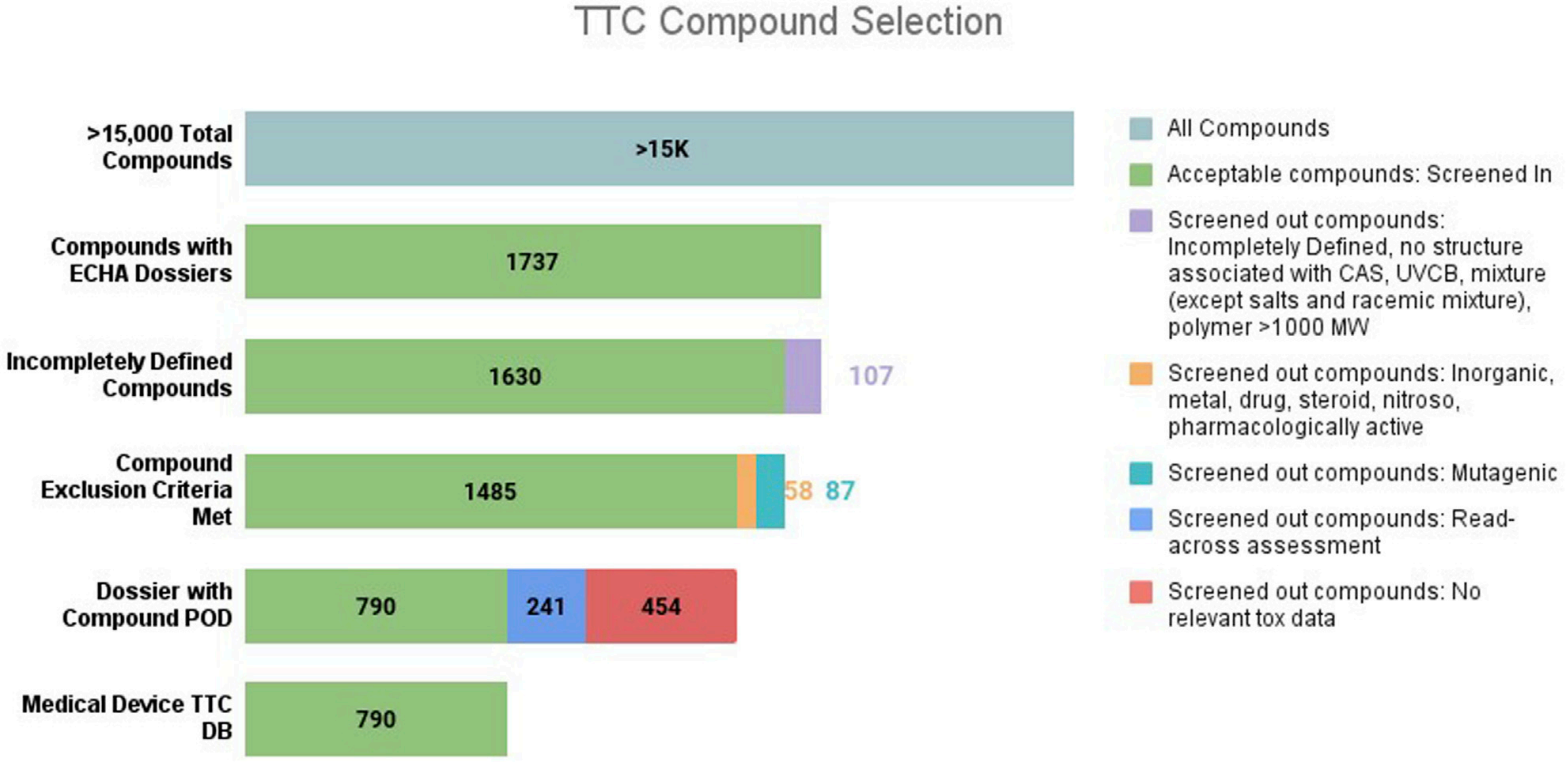
TTC compound selection.

This analysis resulted in a total of 1,485 constituents. From this subset, another 454 constituents were removed due to an absence of reliable repeated dose toxicity studies in the ECHA REACH dossier (i.e., only studies with Klimisch score 3 or 4 were available), and 241 were removed as they relied on the use of read-across NOAEL values (Figure 1).

**FIGURE 2:**
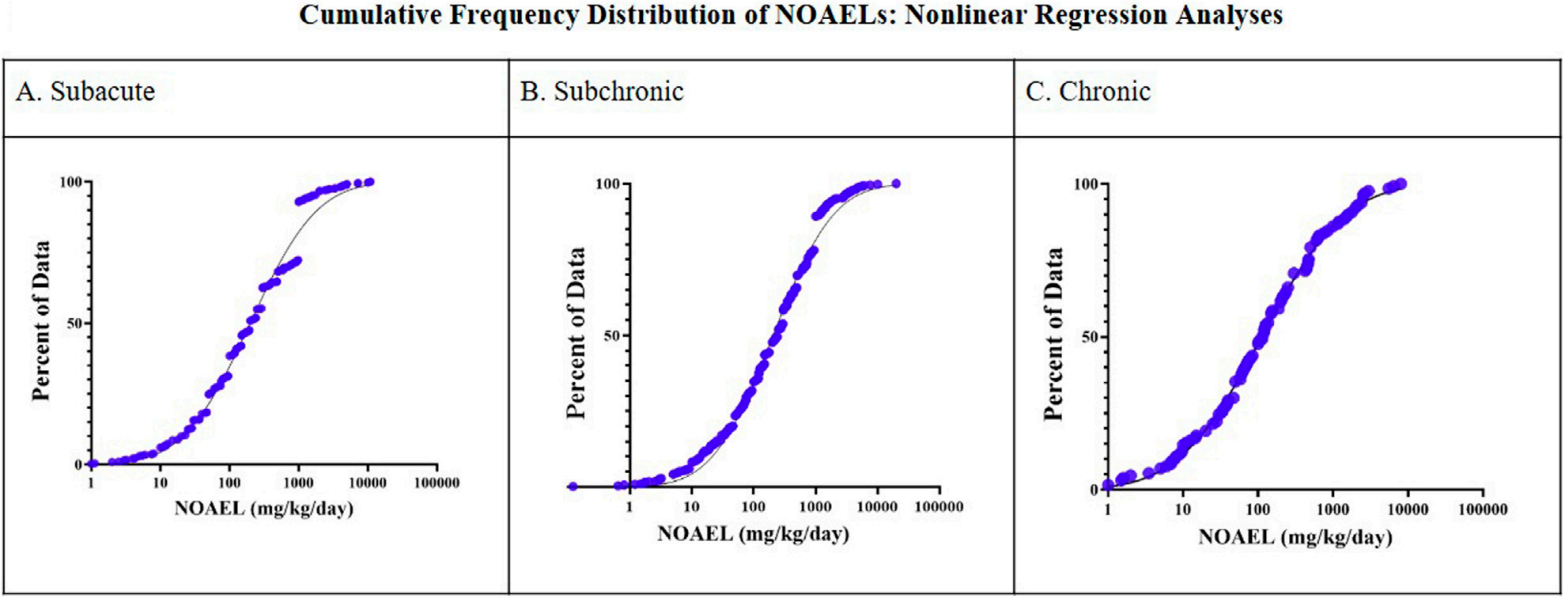
Cumulative frequency distribution of NOAELs: nonlinear regression analysis.

The remaining constituents (n = 790) met all the DB inclusion criteria and were represented by a total of 1,252 NOAELs from subacute (n = 661), subchronic (n = 446), 180-day (n = 43) and 2-year (n = 102) duration studies (Table 2). There were more repeated dose systemic (n = 750) and systemic/developmental and reproductive toxicity (DART) (n = 478) studies than carcinogenicity studies (n = 24). Fewer than 2% of studies included a carcinogenicity endpoint. The rat model was the most studied species across all durations, with less than 1% representation from studies in the hamster, monkey, non-human primate and other species. The number of constituents categorized by Cramer Class I, II and III were 342, 56 and 392, respectively, with low representation by Cramer Class II (7%). The number of subacute, subchronic, 180-day and 2-year studies were 661, 446, 43 and 102, respectively. Overall, there was low representation from 180-day and 2-year studies, supporting the decision to combine these categories when analysis was performed. Since 15 constituents had NOAELs for both 180-day and 2-year durations, the lower NOAEL was chosen for TTC evaluation, resulting in a sample size of 130 NOAELs for the combined duration group (Table 3, Figure 2). Refer to Supplementary File 4 for the MD database used to determine the non-cancer TTC values.

**TABLE 2:** ECHA REACH substances (790 total) that met the inclusion criteria of this study with available oral NOAEL values, stratified by study type, animal species, and Cramer classification.

Stratification	Medical device database					Munro database^a^	
	Subacute	Subchronic	180 days	2 years	Frequency, %^b^	All studies	Frequency, %
Repeat Dose Systemic	258	378	33	81	59.90	233	38.01
DART	403	68	7	—	38.18	180	29.36
Carcinogenicity	—	—	3	21	1.92	200	32.63
Rat	587	409	30	83	88.58	489	79.77
Mouse	21	12	7	10	3.99	90	14.68
Rabbit	45	—	1	—	3.67	31	5.06
Hamster	1	—	—	—	0.08	3	0.49
Dog	6	23	5	7	3.27	—	—
Monkey	—	1	—	—	0.08	—	—
Non-human primate	—	—	—	2	0.16	—	—
Other species	1	1	—	—	0.16	—	—
Cramer Class I	275	186	19	41	41.61	137	22.35
Cramer Class II	49	32	2	5	7.03	28	4.57
Cramer Class III	337	228	22	56	51.36	448	73.08
Cramer Classes I, II, III	661	446	43	102	—	—	—

^a^ Munro, I.C., et al. (1996).

^b^ Frequency percentages were calculated by considering the total number of values (e.g., 1,252 in the medical device database) and the individual values per stratification category.

Note: In the MD, database, a DART, study was inclusive of evaluations with DART, endpoints and those jointly assessing systemic toxicity. A carcinogenicity study included chronic systemic studies if tumor evaluation was an included endpoint. In the Munro Database, a carcinogenicity study was defined as those conducted for a chronic duration.

**TABLE 3:** Lowest fifth Percentile for Cumulative Frequency Distribution: Nonlinear Regression analysis of NOAELs for Each Duration (n = 790 Chemicals).

Study type	Number of values	Lowest 5th percentile (mg/kg/day)
Subacute (14–28 days)	661	11.2
Subchronic (90 days)	446	11.1
Chronic (180 days–2 years)	130	4.1

Note, 15 constituents of the chronic group had both 180-day and 2-year duration NOAELs, resulting in selection of a single lowest NOAEL, and a reduced NOAEL, sample (n = 130).

Considering the application of uncertainty factors of 10 each for inter and intra-species differences (consistent with ISO 10993-17:2023), a modifying factor (MF) of 100 was applied to each value to derive a proposed MD duration-based non-cancer TTC value (Table 4). A TTC of 112 μg/kg/day, 111 μg/kg/day, and 41 μg/kg/day is applicable for MD constituent exposure duration that is ≤ 1–30 days, 31–365 days, and ≥ 366 days, respectively.

**TABLE 4:** Medical device non-cancer TTC values and comparison to established TTC values.

Device categorization (ISO 10993-1)	Limited (≤24 h)	Prolonged (>24 h to 30 days)	Long-term (>30 days)	
Patient/User constituent exposure duration (ISO 10993-17:2023)	≤ 1 day	2–30 days	31–365 days	≥ 366 days
MD Non-Cancer TTC (µg/kg/day)	112	112	111	41
Munro 1996 Cramer Class I TTC (µg/kg/day)	NA	NA	NA	30
Munro 1996 Cramer Class II TTC (µg/kg/day)	NA	NA	NA	9
Munro 1996 Cramer Class III TTC (µg/kg/day)	NA	NA	NA	1.5

NA: not applicable.

Note: Proposed TTC, Values are based on the lowest fifth percentile NOAEL ÷ MF, of 100.

### Chemical space analysis

**FIGURE 3:**
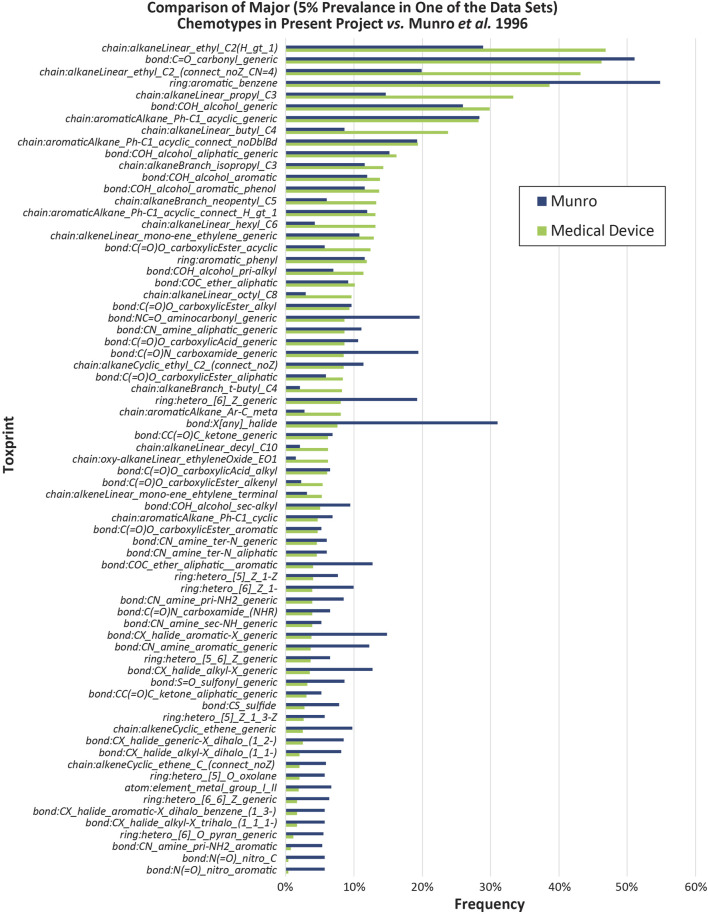
Comparison of Major (5% Prevalance in One of the Data Sets) Chemotypes in Present Project vs. Munro et al. (1996).

**FIGURE 4:**
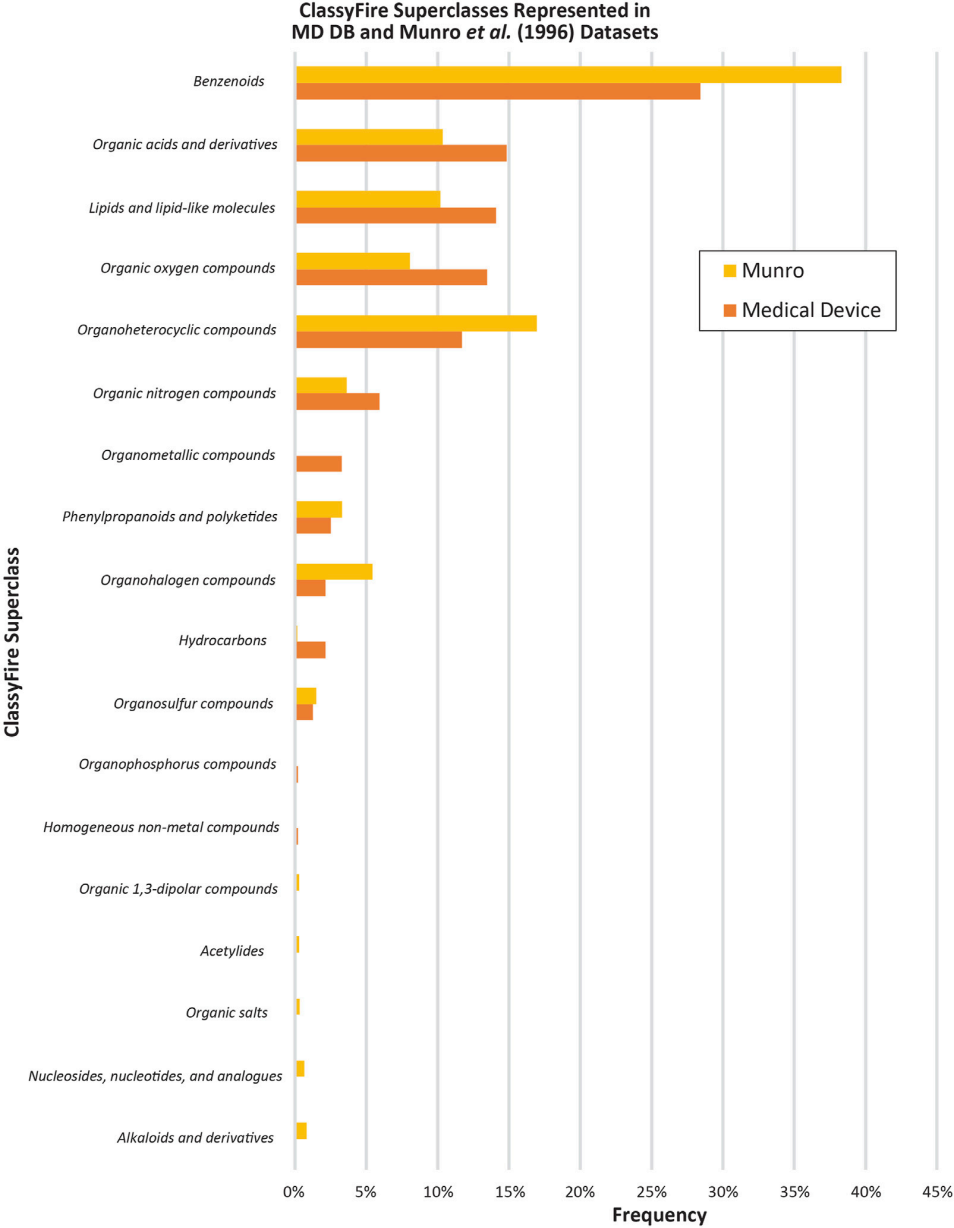
ClassyFire superclasses represented in MD DB and Munro et al. (1996) datasets.

The dataset has diverse constituent types and is pertinent to MDs with a history of market approval. ChemoTyper defined 346 TTC ToxPrint chemical substrings for the MD DB. There was ≥1% frequency for 158 types of fingerprints and ≥5% frequency for 40 fingerprints. Some of the fingerprints representing the lowest frequency (n = 1) in the MD DB included heterocyclic rings, aromatic halides, and amino acids. Figure 3 shows the chemical substructure comparison with the Munro DB. Some notable differences in the two datasets include a higher representation of the ring:aromatic_benzene fingerprint (55%–38%) and bond:X[any]_halide (31%–8%) in the Munro DB, and higher frequency of multiple chain:alkaneLinear fingerprints in the MD DB (chain:alkaneLinear_ethyl_C2(H_gt_1), 47%–29%, chain:alkaneLinear_ethyl_C2_(connect_noZ_CN = 4), 43%–20%, and chain:alkaneLinear_propyl_C3, 33%–15% in addition to several others). An analysis similar to the ChemoTyper analysis was performed using ClassyFire. Figure 4 shows the results of both the MD DB and Munro DB evaluated by SuperClass (as defined by ClassyFire).

**FIGURE 5:**
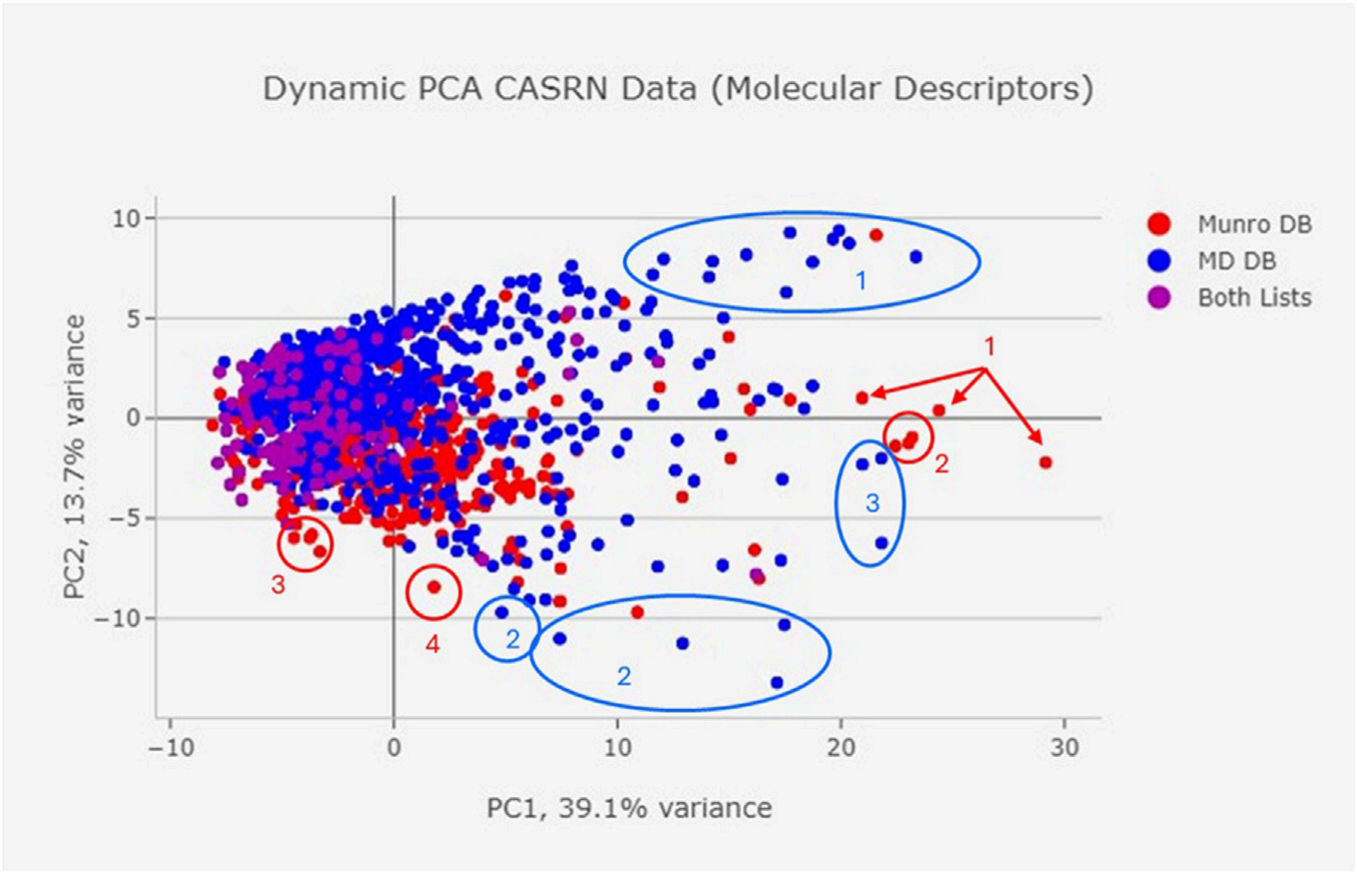
Dynamic PCA CASRN data (molecular descriptors).

From the dynamic PCA plot based on molecular descriptors, the chemicals in the MD DB occupy a wider chemical space (i.e., greater variability among the molecular descriptors) than the Munro database (Figure 5). There is a large area of overlap between the two DBs, but there are outliers within the chemical spaces. Certain types of constituents have greater representation in the MD DB compared to the Munro DB, such as chemicals with long alkyl chains containing central electronegative heteroatoms (e.g., oleyl palmitide, dioctadecyl disulfide; Figure 5, blue oval 1) aromatic colorants (e.g., pigment red 149; Figure 5, blue oval/circle 2); and antioxidants (e.g., Hostanox03, Doverphos 9228; Figure 5, blue oval 3). The Munro DB contains a greater number of carbohydrate-containing chemicals (e.g., cyclodextrin, beta-; Figure 5, red arrows 1); avermectins (Figure 5, red circle 2); and polyhalogenated constituents (decabromodiphenyl oxide, pentachloronitrobenzene; Figure 5, red circles 3 and 4).

## Discussion

As approaches for the toxicological risk assessment of MDs continue to evolve, there is currently no FFP non-cancer TTC value for the assessment of MD constituents that lack toxicological data. To address this gap, this work aimed at deriving MD duration-based non-cancer TTC values following an approach similar to that used by Munro et al., in 1996 to derive non-cancer TTC values based on Cramer Class stratification. To achieve this goal, a MD constituent database was created, its unique chemical space was characterized, and non-cancer MD TTC values were derived for different exposure durations.

### Characterization of the medical device database

A toxicological database comprised of MD constituents is provided in an open-access format for the first time (Supplementary File 4). The DB contains a collection of toxicological information on chemicals specific to the industry provided in an easily mutable format to suit end-user tooling for toxicological risk assessment projects. The contents include reliable toxicological data from four duration categories (subacute, subchronic, 180-day and 2-year) and a single NOAEL selected per duration, obtained from the critical endpoint reported in systemic toxicity, reproductive, developmental or carcinogenicity studies (Table 2). The DB has a high number of subacute and subchronic toxicity studies (n = 1,107), whereas chronic and lifetime studies had lower numbers. Repeat dose systemic toxicity and systemic/DART themed studies were present at greater numbers (98%) than carcinogenicity studies (2%), as would be expected for a dataset containing non-genotoxic constituents.

The MD DB contains a high number of Cramer Class I and III constituents (n = 342 and 392, respectively), and a significantly lower number of Class II (n = 56). The combined constituents of Class I and III (∼93%) make up a majority of the dataset. The project utilized a single QSAR platform to generate Cramer Class predictions and did not further explore other *in silico* software or validate with manual expert judgement to confirm the Cramer predictions. Cramer Class level can be generated using a variety of QSAR models and the prediction can be variable depending on the *in silico* tool used. The reliability of QSARs has been reported for several *in silico* tools based on non-MD datasets (Roberts et al., 2015; Patel et al., 2020). These studies indicate that prediction discordance is attributable to differences in interpretation of the Cramer rules in the programing logic associated with certain chemical subclasses. To improve confidence of the prediction, refer to OECD for general validation principles for QSAR. Use of the ECHA REACH toxicological data repository resulted in a significant number of constituents not meeting the first criterion for inclusion: availability of toxicology data. More than 13,000 constituents did not have an ECHA REACH dossier with toxicological information, and another ∼700 constituents used read-across or weight of evidence NOAELs, thereby resulting in exclusion from analysis (see Figure 1). This outcome aligns with the ECHA REACH toxicological data repository submission process in that it does not require chemical-specific toxicity data be generated for an application.

Given that the Munro DB is the standard for the non-cancer TTC framework, comparison is fitting for a project that seeks to determine the fitness of the tiered TTC scheme applied to a specific application such as MD constituents. The low representation of Class II (7%) in the MD DB is aligned with the Munro DB (4.6%) for this category. The MD DB contains a similar percentage of Class I (43%) and III (∼50%) constituents, whereas the Munro DB had a higher percentage of Class III (73.08%) constituents. The predominance of Class III constituents in the Munro DB generated several independent retrospective analyses (More et al., 2019; Leeman et al., 2014; Nelms et al., 2019; Patlewicz et al., 2022; Kroes et al., 2004).

The Munro DB and MD DB share some common chemicals; however, the MD constituent repository contains distinct constituents that were not included in the Munro DB. The differences in DB composition are confirmed in the Chemotyper and Classyfire analyses (Figures 3, 4), as well as the visualized dynamic PCA results of Figure 5, where outlying data points indicate unique chemical structures that are represented in either only the MD DB or only the Munro DB. Chemicals in the Munro DB that are not represented in the MD DB include cohorts of concern (e.g., polybrominated diphenyl ether; Figure 5, red circle 4), as well as other polyhalogenated aromatics (Figure 5, red circle 3), and pharmaceuticals (e.g., avermectins; Figure 5, red circle 2). The absence of these compounds is not considered to adversely impact the representation of the MD DB chemical space because MDs are not expected to contain these constituents. Except for carbohydrate-containing constituents (Figure 5, red arrows 1) and polyhalogenated constituents (decabromodiphenyl oxide, pentachloronitrobenzene (Figure 5, red circles 3 and 4), the MD DB chemical space encompasses the chemical space of the constituents in the Munro DB.

The TTC derivation described here excluded extractables based mainly on the exclusion categories in the most recent EFSA guidance (More et al., 2019). These categories include potent genotoxic carcinogens, steroids, and substances with a high bioaccumulative potential, such as polyhalogenated-dibenzodioxins, as well as inorganic substances and metals that are not well represented in the current TTC databases, such as that from Munro. Additional categories include proteins, nanomaterials, radioactive substances and high molecular weight polymers. In addition to these categories, substances with known or suspected pharmacological activity were excluded based on the presumption of their potency. This additional exclusion is consistent with that of Kroes et al. in the application of the TTC to cosmetic ingredients (Kroes et al., 2007). The exclusion list here aligns with the most current thinking with respect to substances that should be excluded from the application of the TTC.

The most common excluded category found in the DB was inorganic substances, which is consistent with the use of various inorganic materials as additives or processing aids in MD materials, such as polymers and metals. The second most common category of excluded constituents was that of compounds with pharmacological activity. Some of these constituents, such as steroids, may be components of drug-device combination products. The addition of steroids to some MDs has been common practice for several decades. Other pharmacologically active constituents could be present as contaminants or could have been misidentified.

The lack of certain exclusion categories in the DB may reflect the absence of these constituents in MDs or the inability of extractables studies to report these constituents. Certain exclusion categories are not expected in MDs due to their known sources. For example, aflatoxins are the product of specific species of molds that can contaminate foodstuffs (Rushing and Selim, 2019). The one nitroso constituent identified in the database suggests that at least some of these chemicals are detectable by extractables methodology.

### Non-cancer duration-based TTC values for the evaluation of medical device constituents

Munro et al. (1996) derived chronic TTC values from a database of 691 organic chemicals from industry, pharmaceutical, food, environment, agriculture, and consumer products using NOELs from oral chronic and subchronic toxicity studies. Chemicals were categorized manually using the decision tree of Cramer et al. (1978), which resulted in 137 Class I, 28 Class II, and 448 Class III chemicals (Table 2). To derive TTC values a safety factor of 100 was applied to the fifth percentile NOEL value of each class (Table 4). No chemicals, such as organophosphates, were excluded based on their potency of adverse effects. When comparing the ≥366 days MD non-cancer TTC value to the Munro lifetime TTC values, the MD TTC value is higher, reflecting differences in the data set used to determine the lowest fifth percentile [i.e., Munro’s Class III dataset had a bias toward higher potency NOAELs such as organophosphates and carbamates (n = 40 of 408) (Leeman et al., 2014)], such that a reanalysis removing organophosphates from Class III increased the fifth percentile NOAEL by a factor 2 (Munro et al., 2008). Furthermore, Munro lifetime TTC values applied a duration-based adjustment factor for NOAELs from subchronic studies. The results from this study demonstrate the lifetime TTC values by Munro et al. (1996) are conservative for the evaluation of non-genotoxic chemicals derived from a refined dataset of MD constituents. This paper expands upon the non-cancer TTC values used for lifetime exposure by proposing non-cancer TTC values derived for LTL exposures. The derived TTC values were 112 (≤1–30 days), 111 (31–365 days), and 41 μg/kg/day (≥366 days). Considering the criteria used to select the POD was inclusive of DART studies and contributed to a large subset of the overall NOAELs (from Table 2, DART NOAELS represented a frequency of 38% of the total NOAELs), these non-cancer MD TTC values are considered protective of developmental and reproductive toxicity endpoints in addition to non-cancer systemic toxicity. However, these MD non-cancer TTC values are not applicable for constituents that fall into one of the excluded chemical classes described above.

An immediate benefit from implementation of the LTL non-cancer TTC values is their use in Margin of Safety (MOS) determination. The application of these values allows the selection of duration-based threshold values that correspond to the exposure durations for constituents described in ISO 10993-17:2023. This standard describes in detail the toxicological risk assessment process for MD constituents, including the use of the TTC when the constituent’s specific toxicological information is inadequate to derive a tolerable intake. Typically, the lifetime TTC values would be applied for all durations regardless of the exposure duration category, which can lead to overly conservative MOS values for short exposure durations. The appropriate use of these duration-based TTC values for LTL exposure durations should result in more realistic MOS values and an improved estimate of toxicological risk. Application of duration-based TTC values specifically derived from data on MD constituents allows the toxicologist to evaluate non-cancer LTL endpoints (e.g., acute, subacute and subchronic systemic toxicity) and more appropriately characterize risk, rather than defaulting to a lifetime Cramer Class TTC that may overestimate potential risk from LTL exposure.

TTCs and similar threshold values for LTL exposures have been published for non-cancer endpoints (Kenyon et al., 2024; Buist et al., 2016; Bercu and Dolan, 2012). Both Buist et al. and Bercu and Dolan derived LTL thresholds by applying factors of 3 and 10 to longer-term thresholds for pesticides and pharmaceuticals, respectively. In neither case were the short-term thresholds derived directly from data from subacute and subchronic toxicity studies as was done here. Kenyon et al. did not derive TTCs *per se*, but calculated fifth percentiles of NOAELs from subacute, subchronic, and chronic studies from large datasets of chemicals, including pharmaceuticals and pesticides. A comparison of fifth percentile NOAEL values calculated here and by Kenyon et al. shows that the values determined herein are approximately 5-, 18-, and 41-fold higher for the ≤1–30 days (subacute), 31–365 days (subchronic) and 366 days (chronic) categories, respectively. These differences are likely due to differences in methodology and the chemical dataset, which Kenyon et al. found to have significant overlap with marketed drugs.

In a previous work from Patlewicz et al., a chronic non-cancer TTC value of 1.7 μg/kg/day was derived for MD extractables (Patlewicz et al., 2022). It is acknowledged that this value is significantly lower than the MD TTC of 41 μg/kg/day proposed here for the chronic duration of contact. Nevertheless, awareness of the differences in the methodology applied can explain this discrepancy, notably the small size of the dataset (n = 143), the source of the dataset (i.e., ELSIE DB representing only constituents released from pharmaceutical packaging and drug delivery systems), the use of uncertainty factors to extrapolate subacute and subchronic NOAEL values to chronic values (i.e., 6 and 2, respectively) and the bootstrapping of the log10(NOAEL), applied 1,000 times, that resulted in a median TTC percentile of 1.697 μg/kg/day but with a very wide 95th confidence interval [0.25 μg/kg/day, 16.67 μg/kg/day]. These results highlight the importance of evaluating the methodology applied when comparing TTC values. The value proposed by Patlewicz et al., while being the first of its kind for MDs, is not as robust in methodology nor sample size when compared to that derived in this paper to meet the necessary rigor for establishment of a threshold value.

Finally, the collection of toxicity data for MD constituents revealed that data from routes of exposure other than oral (intravascular, parenteral, or dermal) are less frequent. The duration-based MD TTC values derived herein resulted from a large dataset of several hundred MD chemicals using a highly conservative approach that selected the most relevant POD for each constituent, determination of the fifth percentile of the respective duration dataset, and application of a modifying factor of 100 to account for inter and intra-species differences. Consideration of absorption, distribution, metabolism and excretion (ADME) properties to account for other routes of exposure has been proposed by other researchers (Ellison et al., 2021; Masuda-Herrera et al., 2022; Masuda-Herrera et al., 2023; Partosch et al., 2015) and warrants consideration as a future endeavor for this dataset. The TTC values proposed in this paper are based on oral routes of exposure. If desired, application of additional uncertainty factors to account for route-to-route extrapolation should be considered, based on recommendations in ISO 10993-17:2023.

In conclusion, the MD DB provides insights into the previously unspecified MD constituent space. The chemicals are provided in the supplement for other researchers interested in expanding or analyzing it to answer new questions aimed at improving the approaches used in the biological evaluation of MDs. In this paper, an MD specific database was used to derive non-cancer duration-based MD TTC values. These MD TTC values are expected to support risk assessors with thresholds for evaluating data-poor MD constituents and provide LTL (i.e., duration based) non-cancer TTC values that can be used in conjunction with the guidance and approaches in ISO 10993-17:2023 and ISO/TS 21726:2019 for the toxicological risk assessment of MD constituents. When compared to TTC values previously derived for non-cancer effects, the MD TTC values described in this paper represent FFP TTC values that can be used for the toxicological risk assessment of data-poor constituents released from MDs. However, consultation with appropriate regulatory authorities is recommended prior to the use of these TTC values in regulatory submissions.

## Data Availability

The original contributions presented in the study are included in the article/Supplementary Material, further inquiries can be directed to the corresponding author.
