# Supplementary material for: Characterization of medical device constituents and development of duration-based non-cancer threshold of toxicological concern values
Source: Front Toxicol. 2025 Jun 4;7:1600127. doi: 10.3389/ftox.2025.1600127 (PMC12175843; doi:10.3389/ftox.2025.1600127)
Supplement: Supplementary file 1 [file Supplementaryfile1.docx]

**Supplemental File 1.**

**List of Chemotypes used to identify extractables from the exclusion list:**

Ring:hetero_[5_5_6]_O_aflatoxin_generic

Bond:N=N_azo_azoxy

bond:N=O_N-nitroso_generic

Bond:C(~Z)~C~Q_haloether_dibenzodioxin_1-halo

Bond:C(~Z)~C~Q_haloether_dibenzodioxin_2-halo

Bond:C(~Z)~C~Q_haloether_dibenzodioxin_dichloro_(2_7-)

Bond:C(~Z)~C~Q_haloether_dibenzodioxin_tetrachloro_(2_3_7_8-)

Bond:CX_halide_aromatic-X_biphenyl

Bond:CX_halide_aromatic-X_dihalobenzene_(1_2-)

Bond:CX_halide_aromatic-X_dihalobenzene_(1_3-)

Bond:CX_halide_aromatic-X_dihalobenzene_(1_4-)

Bond:CX_halide_generic-X_dihalo_(1_2-)

Ring:fused_steroid_generic_[5_6_6_6]

Bond:CN_amine_aromatic_benzidine

Bond:metal
